# ACERTO PROJECT: IMPACT ON ASSISTANCE OF A PUBLIC EMERGENCY HOSPITAL

**DOI:** 10.1590/0102-672020200003e1544

**Published:** 2021-01-15

**Authors:** Mauricio Adam Feitosa SAMPAIO, Simone Losekann Pereira SAMPAIO, Plinio da Cunha LEAL, Ed Carlos Rey MOURA, Lívia Goreth Galvão Serejo ALVARES, Caio Marcio Barros DE-OLIVEIRA, Orlando Jorge Martins TORRES, Marília da Glória MARTINS

**Affiliations:** 1 Medical Course, Uniceuma, São Luís, MA, Brazil; 2 Nursing Course, Uniceuma, São Luís, MA, Brazil; 3 Postgraduate Program in Adult Health, Federal University of Maranhão, São Luís, MA, Brazil; 4 Course of Medicine, Federal University of Maranhão, São Luís, MA, Brazil

**Keywords:** Clinical protocols, Patient discharge, Postoperative complications, General surgery, Emergency medicine, Protocolos clínicos, Alta do paciente, Complicações pós-operatórias, Cirurgia geral, Medicina de emergência

## Abstract

**Background:**

In Brazil, the goal-based approach was named Project ACERTO and has obtained good results when applied in elective surgeries with shorter hospitalization time, earlier return to activities without increased morbidity and mortality.

**Aim:**

To analyze the impact of ACERTO on emergency surgery care.

**Methods:**

An intervention study was performed at a trauma hospital. Were compared 452 patients undergoing emergency surgery and followed up by the general surgery service from October to December 2018 (pre-ACERTO, n=243) and from March to June 2019 (post-ACERTO, n=209). Dietary reintroduction, volume of infused postoperative venous hydration, duration of use of catheters, probes and drains, postoperative analgesia, prevention of postoperative vomiting, early mobilization and physiotherapy were evaluated.

**Results:**

After the ACERTO implantation there was earlier reintroduction of the diet, the earlier optimal caloric intake, earlier venous hydration withdrawal, higher postoperative analgesia prescription, postoperative vomiting prophylaxis and higher physiotherapy and mobilization prescription were achieved early in all (p<0.01); in the multivariate analysis there was no change in the complication rates observed before and after ACERTO (10.7% vs. 7.7% (p=0.268) and there was a decrease in the length of hospitalization after ACERTO (8,5 vs. 6,1 dias (p*=*0.008).

**Conclusion:**

The implementation of the ACERTO project decreased the length of hospital stay, improved medical care provided without increasing the rates of complications evaluated.

## RESUMO

**Racional:**

No Brasil, a abordagem baseada em metas foi nomeada de Projeto ACERTO e tem obtido bons resultados quando aplicada em operações eletivas com diminuição do tempo de internação, retorno mais precoce as atividades sem incremento de morbimortalidade.

**Objetivo:**

Analisar o impacto do ACERTO na assistência prestada em operações de emergência.

**Métodos:**

Foi realizado um estudo de intervenção em hospital de trauma. Foram comparados 452 pacientes submetidos à operações de emergência e acompanhados pelo serviço de cirurgia geral no período de outubro a dezembro de 2018 (fase pré-ACERTO, n=243) e no período de março a junho de 2019 (fase pós-ACERTO, n=209). Foram avaliados: reintrodução da dieta, volume de hidratação venosa pós-operatória infundido, tempo de uso de catéteres, sondas e drenos, analgesia pós-operatória, prevenção de vômitos pós-operatórios, mobilização precoce e fisioterapia.

**Resultados:**

Após a implantação do ACERTO houve reintrodução mais precoce da dieta, foi atingido o aporte calórico ideal mais precocemente, retirada mais precoce da hidratação venosa, maior prescrição de analgesia pós-operatória, de profilaxia de vômitos pós-operatórios e maior prescrição de fisioterapia e mobilização precoce em todos (p<0,01); na análise multivariada não houve alteração nas taxas de complicações observadas pré e pós-ACERTO (10,7% vs. 7,7% (p*=*0,268) e houve diminuição do tempo de internação pós-ACERTO (8,5 vs. 6,1 dias (p*=*0.008).

**Conclusão:**

A implantação do projeto ACERTO diminuiu o tempo de internação hospitalar, melhorou a assistência médica prestada sem incremento das taxas de complicações avaliadas.

## INTRODUCTION

The ACERTO project (ACEleration of Postoperative Total Recovery) is based on the ERAS protocol (Enhanced Recovery After Surgery) in which care is guided by daily goals based on evidence-based medicine^21^ ^,^ ^24^ ^,^ ^25^. The implementation of this form of care has shown a significant reduction in postoperative complications and reduced the length of hospital stay by 30-50%, and today it is adopted in more than 20 countries as the ideal form of surgical assistance^12^.

The ERAS protocol includes multimodal and multidisciplinary assessment of 15 to 20 items that cover the pre, trans and postoperative period. Isolated, these items have little clinical expression, but together they contribute significantly to the reduction of post-surgical stress, surgical complications, pain, recovery time and length of hospital stay^10^ ^,^ ^12^.

The ERAS project started in the 1990s by Henrik Kehlet as a patient-centered fast-track protocol with the cooperation of the medical, nursing, nutrition and psychology staff. It aimed to reduce surgical stress, surgical complications and accelerate postoperative recovery^6^. It was applied primarily in Europe to accelerate postoperative recovery in patients undergoing colorectal operations. The results demonstrated are reproducible worldwide and show a reduction in the length of hospital stay after its implantation, as well as being associated with a lower number of complications^11^.

The goal-based approach contributes to the reduction of complications in colorectal operations, decreases hospital costs and has been investigated in other surgical sites regarding the effectiveness and possible associated risks. In a systematic review investigating the use of the protocol in high abdominal operations, a decrease in morbidity from 22% to 14% (p=0.017) and hospital stay from 7.5 to 5.7 (p=0.019) was observed without statistical differences in mortality and readmissions^17^.

In Brazil, the ERAS protocol was adapted, received the name ACERTO and was first implemented at the Júlio Muller University Hospital, Cuiabá, MT, Brazil with reduced hospital stay, use of blood products, decreased cases of surgical site infection, complications operative and deaths. It has been validated in multiple operations ranging from colorectal, cardiac and even oncological operations, where a decrease in the volume of intravenous fluids has been observed, shorter hospital stay when preoperative fasting has been reduced^21^.

The ACERTO project covers the assessment of preoperative factors such as patient information, nutritional therapy, decreased fasting^3^; also transoperative factors such as rational use of catheters, probes, drains and the rational use of prophylactic antibiotics; and finally, postoperative factors such as analgesia, prevention of nausea, vomiting and ultra early mobilization. The intervention points were adapted to the epidemiological reality of Latin America^21^.

Some points of the ACERTO project involve preoperative care and are not accessible most often to patients in urgent and emergency units; however, some fundamental factors in the management of these patients and which have proven statistical relevance can be verified in the trans and postoperative periods^5^. Aiming to focus on the assistance provided during the trans and postoperative period in order to avoid unnecessary measures and the patient to return to the usual physiological conditions as soon as possible, the following were included in this evaluation: early start of the diet, restrictive venous hydration, rational use devices (catheters, probes and drains), prophylaxis of postoperative nausea and vomiting, postoperative analgesia, early mobilization and physiotherapy.

This study aims to assess whether the ACERTO project measures applied in a surgical ward of an urgency and emergency hospital could result in more efficient care and reflect in reducing the length of hospital stay without adding morbidity and mortality to patients.

## METHODS

This study started after the approval of the ethics committee in research with human beings of the CEUMA University under the CAAE protocol 2.586.802, and it was registered with the Brazilian Registry of Clinical Trials under registration number RBR-9tzrzx. It is a type of intervention before and after, where 452 patients were evaluated, submitted to urgent and emergency surgery in a public trauma hospital in São Luís, MA, Brazil. The observation took place in two phases: an initial one from October to December 2018, before the implementation of the ACERTO project, and another from March to June 2019 after the implementation of the ACERTO project.

Service meetings were held with the participation of assistant surgeons, nurses, physiotherapists, nursing technicians and nutritionists. At these meetings, the following topics were addressed: perioperative nutrition; perioperative venous hydration; rational use of probes, catheters and drains; postoperative analgesia; prophylaxis of nausea and vomiting; ultra early mobilization and physiotherapy. The process generated assistance protocol used as an ideal treatment method and facilitated through a conducting diagram and the changes implemented are described in Table 1. Clinical audits were carried out to verify the teams’ adherence to the new recommended conducts.

**TABLE 1:** Conducts applied in the surgical ward before and after applying the ACERTO Project

	Traditional care	ACERTO Project
Nutrition	Release of diet after release of flatus	• Ano-orifical, biliary, herniorraphies and correlated: start a liquid diet on the same day of the operation. • Anastomosis operations: start a diet on the 1st PO
Venous hydration	Prescribe 40 ml/kg	• Extraperitoneal operations: do not prescribe HV in the PO • Consider suspension of HV in the 1st PO • When necessary, do not exceed 30 ml/kg
Drains	According to the surgeon’s preference	• Routine drainage only in esophageal operations • drains must be removed within 72 h (unless there are clinical contraindications)
Analgesia	It was not routinely prescribed	• Routine analgesia prescription • Use associations if necessary
Prophylaxis of vomiting	It was not prescribed	• Start with Plasil® • If you fail to associate 5HT3 antagonist • If you fail, triple therapy with the two above + dexamethasone + promethazine
Ultra-early mobilization	It was not routinely prescribed	• Ambulation and stay out of bed for 2 h on the day of the operation and 6 h on the subsequent days • Respiratory and motor physiotherapy

HV=venous hydration; PO=postoperative period

### Statistical analysis

The research data were evaluated using the IBM SPSS Statistics 20 (2011) statistical program. Initially, descriptive statistics of continuous variables were made, that is, the minimum, maximum, median, mean and standard deviation were estimated, then they were evaluated for normal distribution using the lilliefors test and as they presented normal distribution, they were assessed by the parametric Student’s t test. Then, to assess the association of sociodemographic and clinic-surgical variables in relation to the two moments (before and after), the non-parametric chi-square test of independence (x2) was performed. In all tests, the level of significance applied was 5%, that is, it was considered significant when p<0.05.

## RESULTS

There was similarity between the groups studied in the clinical and epidemiological characteristics, with a slight difference in terms of gender and origin (Table 2).

**TABLE 2:** Sociodemographic characteristics in the period before and after the implementation of the ACERTO Project

Socio-demographic	Before		After		
	n=243	%	n=209	%	p
Genre					
Male	172	70.8	127	60.8	0.025
Female	71	29.2	82	39.2	
Marital status					
Not married	128	52.7	99	47.4	0.476
Married	61	25.1	51	24.4	
Stable union	43	17.7	49	23.4	
Divorced	3	1.2	1	0.5	
Widower	8	3.3	9	4.3	
Age range					
<20	12	4.9	19	9.1	0.245
20-29	66	27.2	54	25.8	
30-39	66	27.2	68	32.5	
40-49	34	14.0	30	14.4	
50-59	35	14.4	18	8.6	
60-69	13	5.3	7	3.3	
70-79	13	5.3	8	3.8	
> 79	4	1.6	5	2.4	
Profession					
Farmer	38	15.6	26	12.4	0.738
Student	28	11.5	21	10.0	
Self-employed	20	8.2	17	8.1	
Domestic	20	8.2	16	7.7	
Retired	17	7.0	11	5.3	
Bricklayer	10	4.1	17	8.1	
Driver	9	3.7	6	2.9	
Fisherman	7	2.9	13	6.2	
Mechanical	7	2.9	1	0.5	
Salesman	6	2.5	9	4.3	
Vigilant	5	2.1	3	1.4	
Teacher	3	1.2	4	1.9	
General Services	3	1.2	4	1.9	
Electrician	3	1.2	3	1.4	
Painter	2	0.8	2	1.0	
Nursing technician	2	0.8	2	1.0	
Carpenter	2	0.8	1	0.5	
Businessman	2	0.8	1	0.5	
Seafood	2	0.8	1	0.5	
Barber	1	0.4	3	1.4	
Fitter	1	0.4	3	1.4	
Dressmaker	1	0.4	2	1.0	
Others	54	22.2	43	20.6	
Origin					
Capital	156	64.2	114	54.5	0.012
Interior	83	34.2	95	45.5	
Other states	4	1.6	0	0.0	
Total	243	100.0	209	100.0	

There was a slight statistical difference regarding the surgical procedures performed comparing the two phases (pre- and post-ACERTO), but with a predominance of more serious injuries (laparotomy for multivisceral injuries) in the period after the implementation of the protocol (Table 3).

**TABLE 3:** Surgical procedures performed before and after the implementation of the ACERTO Project

Clinical/Surgical	Before		After		
	n=243	%	n=209	%	p
Surgical procedure					
Appendectomy	81	33.3	64	30.6	0,038
Debridement	37	15.2	29	13.9	
Reconstruction of wounds	20	8.2	13	6.2	
Abscess drainage	19	7.8	22	10.5	
Laparotomy (multivisceral lesions)	10	4.1	16	7.7	
Oophorectomy	9	3.7	11	5.3	
Inguinal herniorrhaphy	8	3.3	10	4.8	
Hepatorraphy	6	2.5	2	1.0	
Fasciotomy	5	2.1	5	2.4	
Enterectomy	5	2.1	3	1.4	
Enteroanastomosis	5	2.1	3	1.4	
Gastrorraphy	5	2.1	3	1.4	
Inguinal hernioplasty	5	2.1	0	0.0	
Cholecystectomy	4	1.6	4	1.9	
Acute obstructive abdomen	4	1.6	2	1.0	
Bartholinectomy	4	1.6	2	1.0	
Umbilical herniorrhaphy	4	1.6	1	0.5	
Incisional herniorrhaphy	3	1.2	0	0.0	
Splenectomy	2	0.8	1	0.5	
Finger amputation	2	0.8	0	0.0	
Colostomy	2	0.8	0	0.0	
Closed chest drainage	2	0.8	0	0.0	
Salpingectomy	1	0.4	12	5.7	
Others	16	6.6	6	2.9	
Total	243	100.0	209	100.0	

There was a decrease in the time of reintroduction of the diet, with 76.6% of the patients starting a diet in the first post-operative period, 42% of the pre-ACERTO group (p<0.001). The ideal caloric intake was reached in the first two days in 84.2% after the ACERTO vs. 69.1% in the pre-ACERTO group (p=0.002, Table 4). 

**TABLE 4:** Comparison of the intervention on the diet in the periods before and after the ACERTO project

Nutrition	Antes		Depois		
	n=243	%	n=209	%	p
Diet reintroduction day					
1^st^ day	102	42.0	160	76.6	< 0.001
2^nd^ day	108	44.4	34	16.3	
3^rd^ day	21	8.6	8	3.8	
=4^th^ day	12	5	7	3.3	
Ideal caloric intake achieved					
Until the 2^nd^ day	168	69.1	176	84.2	0.002
3^rd^ to 4^th^ day	50	20.6	23	11.0	
After the 4^th^ day	25	10.3	10	4.8	
Total	243	100.0	209	100.0	

Venous hydration =30 ml/h was achieved on the first postoperative day in 88.5% of patients after implantation of the protocol vs. 79% before the intervention (p=0.03). There was also a reduction in the time of venous hydration prescribed with 79.4% remaining using hydration for less than three days vs. 70.8% before the implementation of the protocol (p=0.048, Table 5). 

**TABLE 5:** Comparison of fluid prescription before and after the implementation of the ACERTO Project

Venous hydration (HV)	Before		After		
	n=243)	%	n=209	%	p
Day left =30 ml/h					
1	192	79.0	185	88.5	0.030
2	34	14.0	14	6.7	
3	16	6.6	8	3.8	
=4	1	0.4	2	1.0	
Day withdrew HV					
1-3	172	70.8	166	79.4	0.048
4-6	50	20.6	35	16.7	
>6	21	8.6	8	3.8	
Total	243	100.0	209	100.0	

There was a higher frequency of prescription of postoperative analgesia (98.6% vs. 84.0% (p<0.001) as well as vomiting prophylaxis (94.7% vs. 35.8%, p<0.001), of early mobilization (80.9% vs. 4.9%, p<0.001) and physical therapy (80.4% vs. 9.5%, p<0.001) after the introduction of the protocol (Table 6)

**TABLE 6:** Comparison of interventions before and after the implementation of the ACERTO project

ACERTO project interventions	Before		After		p
	n=243	%	n=209	%	
Postoperative analgesia					
Yes	204	84	206	98.6	< 0.001
No	39	16	3	1.4	
Prophylaxis of vomiting					
Yes	87	35.8	198	94.7	< 0.001
No	156	64.2	11	5.3	
Early walking					
Yes	12	4.9	169	80.9	< 0.001
No	231	95.1	40	19.1	
Physiotherapy					
Yes	23	9.5	168	80.4	< 0.001
No	220	90.5	41	19.6	
Total	243	100	209	100	

There was a reduction in the average number of days of hospitalization for patients in the ACERTO group from 8.5 to 6.1 (p=0.008). There were no statistically significant variations in the observed postoperative complications or in mortality (Table 7). 

**TABLE 7:** Rates of postoperative complications before and after the implementation of the ACERTO project

Complications	Before		After		
	n	%	n	%	p
No	217	85.8	193	89.4	0.42
Death	1	0.4	3	1.4	
Fistula	1	0.4	0	0.0	
Reoperation	22	8.7	13	6.0	
Infection	2	0.8	0	0.0	
Readmission	10	4.0	7	3.2	
Total	243	100	209	100	

## DISCUSSION

The implementation of the ACERTO project requires continuous auditing to observe unwanted consequences such as an increase in the readmission rate. In a recent meta-analysis, the application of fast-track protocols decreased the rates of post-surgical complications^8^. This fact was confirmed in a prospective study by Wood et al.^28^ that followed patients in the first 30 postoperative days and in a Spanish series^20^ that followed patients who underwent laparoscopic operations and evaluated complications up to the period of 180 days. The most commonly observed gain was in reducing the length of hospital stay as observed in the retrospective Cohort by Wisely et al.^27^, in the systematic review by Paduraru et al.^18^ and in a recent study comparing patients undergoing gastric bypass^1^.

In this study, a decrease was observed both in the time of reintroduction of the diet and in the day of the adequate nutritional supply. This factor contributes to the patient’s lower catabolism and, also, to the possibility of earlier discharge as stated in the ACERTO^6^ nutritional intervention guidelines and in the international literature as an ideal form of care^22^. These data corroborate with the recent literature in which patients with earlier return to their usual diet are discharged earlier^13^ ^,^ ^15^.

There was a reduction in the volume of venous hydration, the amount of volume prescribed, as well as a reduction in the length of time the venous hydration remains during hospitalization. Restrictive fluid replacement has been shown to be superior in the treatment of surgical patients, decreasing cytotoxic edema that worsens oxygenation and tissue recovery, decreasing the adynamic ileum and preventing cardiopulmonary complications in more susceptible patients^23^. In a recent systematic review Miller^16^ talks about the suspension of venous hydration as soon as possible and the search for zero water balance as an ideal form of care.

Postoperative analgesia was optimized with the implementation of the ACERTO project, with adaptation to the protocol in 98.6% of patients. Analgesia allows better mobilization, as well as increases the feeling of well-being, providing early discharge. In the systematic review by Wick et al.^26^, the importance of postoperative pain management as well as opioid-sparing strategies was reported.

As for the use of catheters, probes and drains, no statistical difference was observed in this study, perhaps due to the previous effort to abandon this conduct, which was already widespread in the unit. The use of catheters, probes and drains does not decrease the incidence of cavity collections^29^ and increases the incidence of pleuropulmonary complications^19^.

Prophylaxis of vomiting was adequate in 94.7% of patients. This point allows greater tolerance of the diet, decreases the patient’s malaise, increases his confidence in the recovery process and, consequently, decreases hospital costs^7^.

Early mobilization and physiotherapy were prescribed to more than 80% of patients and encouraged by the entire multidisciplinary team. The study by Boden et al.^2^ talks about the beneficial effects of early mobilization and physiotherapy in patients undergoing upper abdomen operations, such as improving intestinal transit and decreasing pleuropulmonary complications associated with restriction.

There was a decrease in the length of hospital stay of 8.5 vs. 6.1 days (p=0.008) without statistical increase in morbidity and mortality. This data is in agreement with the current literature when it says that there is a reduction in the length of hospital stay without increasing morbidity to surgical patients submitted to fast-trac protocols^4^ ^,^ ^8^ ^,^ ^14^.

With the same number of beds available, the capacity to treat patients rose from 207 to 258 patients with complete treatment per month. Using the same resources employed, it was possible to treat 24% more patients and there was a virtual gain of 13 beds.

## CONCLUSION

The ACERTO project is feasible and safe for patients undergoing emergency operations at a trauma hospital. They received less postoperative fluids, started their diet earlier, arrived at the ideal caloric intake more quickly, received more postoperative analgesia, increased rates of nausea and vomiting prophylaxis, physiotherapy and early mobilization and were discharged earlier with no statistically significant change in morbidity and mortality rates between the two groups. With the obtained result it was possible to treat 24% more patients with the same resources employed and without adding risks to the patients.
